# Multidisciplinary Care for the Prevention and Treatment of Venous Thromboembolism in Patients with Cancer-Associated Thrombosis (CAT): Impact of Educational Interventions on CAT-Related Events and on Patients’ and Clinicians’ Awareness

**DOI:** 10.3390/life12101594

**Published:** 2022-10-13

**Authors:** Beniamino Zalunardo, Chiara Panzavolta, Paola Bigolin, Adriana Visonà

**Affiliations:** Angiology Unit, San Giacomo Hospital, Castelfranco Veneto, ULSS 2 Marca Trevigiana, 31015 Treviso, Italy

**Keywords:** cancer-associated thrombosis, prevention, treatment, multidisciplinary care, educational interventions, awareness

## Abstract

Cancer is a leading cause of death. Venous thromboembolism (VTE) is an often-overlooked cause of morbidity and mortality in cancer patients that can be readily prevented and treated. Actions are needed to reduce the morbidity and mortality of patients with cancer-associated thrombosis (CAT). There is a need to increase awareness of the impact of CAT on cancer patients’ morbidity and mortality, on their quality of life and to understand the importance of more effective preventions and treatments of VTE in cancer patients. Moreover, it is of great importance to systematically assess the risk of VTE in regard to patients, cancer and treatment-related factors. Unfortunately, there are unmet clinical needs in the prevention and treatment of cancer-associated VTE. In this review, we discuss an action plan to ensure an increased awareness of and education on the issues that need to be addressed in order to improve the provision of appropriate prevention, early diagnosis and effective and safe treatment of VTE to all cancer patients and, ultimately, to reduce morbidity and mortality.

## 1. Introduction

Over the past two decades, in cancer patients, the 12-month cumulative incidence of venous thromboembolism (VTE) has increased three-fold overall and even six-fold in those using chemotherapy or targeted therapy. The 6-month VTE risk for patients with cancer is 12-fold higher compared with the general population and as much as 23-fold higher in patients receiving chemotherapy or targeted therapy [1]. Moreover, patients with VTE and cancer have a higher mortality than patients with VTE alone or with cancer alone [2,3]. 

In cancer patients, risk factors for VTE are previous VTE, distant metastasis, recent surgery and antineoplastic therapy (anti-angiogenic therapy, immunotherapy, use of protein kinase inhibitors, chemotherapy and other therapies targeted) [1].

In an analysis of data on 4466 outpatients on chemotherapy by Khorana in 2007, thrombosis and infection are identified as the second leading cause of death in this patient population (both 9.2%) [4].

The stratification of the thromboembolic risk plays a primary role in identifying patients at high risk and therefore deserving of antithrombotic prophylaxis. The ASCO guidelines actually consider the identification of patients who are most likely to benefit from drug prophylaxis as one of the two foundations of VTE management.

The second foundation of VTE management is the effective treatment of VTE to reduce the risk of VTE recurrence and mortality [5].

Morbidity and mortality in cancer patients can be readily prevented and treated. Actions are needed to reduce the morbidity and mortality of patients with CAT. We considered as a reference a document written by an expert steering group meeting in Belgium “Cancer-associated thrombosis (CAT), a neglected cause of Cancer death: actions needed to increase health outcomes and reduce mortality” [6]. There is a need to increase awareness of:

1. The impact of CAT on cancer patients’ morbidity and mortality, on their quality of life and on the costs of the National Health System (NHS);
2. The means of preventing and treating thrombosis more efficiently and earlier in cancer patients.

Moreover, it is of great importance to systematically assess the risk of VTE in regard to patients, cancer and treatment-related factors.

Unfortunately, there are unmet clinical needs in the prevention and treatment of cancer-associated VTE [7].

The aim of this review is to analyze unmet clinical needs in the prevention and treatment of cancer-associated venous thromboembolism and to review possible pathways for overcoming obstacles in the implementation of guidelines based on articles available in the literature.

We searched MEDLINE for articles published between January 2012 and June 2022 in English and Italian that addressed multidisciplinary care for the prevention and treatment of venous thromboembolism in patients with CAT and the impact of educational interventions on CAT related-events and on patients’ and clinicians’ awareness.

We used the following keywords: cancer-associated thrombosis, prevention, treatment, multidisciplinary care, educational interventions, awareness, unmet clinical needs.

## 2. Pathophysiology of VTE in Cancer Patients

A hypercoagulable condition in cancer patients is known. Common coagulant mechanisms are likely to exist for thrombus formation and tumor growth and progression, demonstrated by altered levels of laboratory markers of the activation of blood coagulation [8].

The pathogenesis of this phenomenon is multifactorial, involving both biological and clinical factors. 

Many clinical factors in cancer patients have been associated with the risk of VTE: those related to the patient such as the history of VTE, advanced age, the presence of comorbidities, those related to cancer such as the location of the tumor, the histological type, the extent of the disease and the time after diagnosis and finally the treatment-related factors, such as surgery, chemotherapy, hormonal therapies, antiangiogenics, CVC placement and transfusions.

Biological factors are equally important: the activation of a direct coagulation pathway, the induction of inflammatory responses, the inhibition of fibrinolytic activity and tumor cell-induced platelet aggregation. In particular, cancer cells release inflammatory cytokines, procoagulant and fibrinolytic proteins and procoagulant microparticles; furthermore, they express adhesion molecules that bind to endothelial cells, platelets and leukocytes and stimulate prothrombotic properties [8].

Much remains to be studied to understand this close relationship between thrombosis and cancer. A better understanding of these interactions is desirable for the future improvement of strategies to prevent and treat VTE.

### 2.1. Primary Prevention of CAT

#### 2.1.1. What Is the Evidence?

Khorana and colleagues developed an easy model for predicting VTE associated with chemotherapy using simple clinical and laboratory variables (Table 1). This risk model was derived from a cohort of 2701 patients and was then validated in an independent cohort of 1365 patients from a prospective registry. The observed incidences of symptomatic VTE over a median follow-up period of 2.5 months in the derivation and validation cohorts were 0.8% and 0.3% in the low-risk category, 1.8% and 2% in the intermediate-risk category and 7.1% and 6.7% in the high-risk category, respectively [9]. The Khorana score can be used to select ambulatory cancer patients at a high risk of VTE for thromboprophylaxis, but unfortunately most events occur outside this high-risk group. In a meta-analysis of 55 studies involving 34,555 ambulatory cancer patients the incidence of VTE in the first six months was 5.0% (95%CI: 3.9–6.5) in patients with a low-risk Khorana score (0 points), 6.6% (95%CI: 5.6–7.7) in those with an intermediate-risk Khorana score (1 or 2 points) and 11.0% (95%CI: 8.8–13.8) in those with a high-risk Khorana score (3 points or higher); 23.4% (95%CI: 18.4–29.4) of the patients with VTE in the first six months had been classified as high risk according to the Khorana score [10].

Moreover, the Khorana score is not sufficient to predict the risk of VTE in some types of cancer (lung, pancreas), while it seems to be a good predictor of all-cause mortality in lung cancer patients [11,12,13]. A recent meta-analysis, which included five randomized controlled trials, confirmed the data of a previous meta-analysis, namely that pharmacological antithrombotic prophylaxis significantly reduced the risk of VTE compared with a to placebo without increasing the bleeding risk (NNT 11.9). There was no difference between parenteral anticoagulants and oral anticoagulants, nor between prophylactic and supra-prophylactic doses [14]. Therefore, guidelines recommend the pharmacological antithrombotic prophylaxis of VTE with low-molecular-weight heparins (LMWHs) in outpatient patients with locally advanced or metastatic pancreatic cancer treated with systemic antineoplastic therapy and who have a low risk of bleeding [15]. A recent meta-analysis by Becattini confirmed that with antithrombotic prophylaxis there was a significant reduction of VTE in patients with lung cancer (OR 0.42, 95% CI 0.26–0.67) and with pancreatic cancer (OR 0.26; 95% CI 0.14–0.48) [16].

Several other retrospective and prospective studies have further validated the Khorana score, although the incidences of VTE vary between studies due to variations in patient selections and follow-up periods. Other variations of the Khorana score were made to improve the risk assessment, such as the Vienna CATS Score, the PROTECHT score and the CONKO score [17].

The panel of the Italian Society of Hematology verified the limited value of the Khorana score in patients with hematologic neoplasms and therefore formulated a specific recommendation to select specific risk scores for the patients’ pathology, if available [18]. Studies in patients with multiple myeloma have shown high incidences of VTE [19]. The ASH guideline panel suggested using low-dose aspirin, fixed low-dose VKA or LMWH for multiple myeloma patients receiving lenalidomide-, thalidomide- or pomalidomide-based regimens [20]. 

If Khorana score struggles to predict VTE for all types of cancer, and one should be looked for each type of cancer, efforts to use clinical risk factors not included in a score as a stratification strategy risk have failed. Two examples of this failure are the two large studies on prophylaxis with semuloparin or nadroparin in cancer patients receiving chemotherapy, SAVE-ONCO and, respectively. Patients had solid tumors with locally advanced or metastatic disease extension and high thromboembolic risk. Despite this, the incidence of events in the placebo group was low (3.4% in the SAVE-ONCO and 3.9% in the PROTECHT). This resulted in a high NNT (45 in the SAVE-ONCO and 53 in the PROTECHT). This means that we would have to give antithrombotic prophylaxis to about fifty patients to be able to prevent a venous thromboembolic episode [21].

The purpose of risk stratification is the selection of patients for antithrombotic prophylaxis. Results of the randomized trials show that outpatient prophylaxis is safe and effective, but the risk–benefit ratio could significantly improve if high-risk patients can truly be identified. Two recent analyses suggest that the risk score improves the risk–benefit ratio of antithrombotic prophylaxis in outpatients. A post hoc subgroup analysis of the PROTECHT study was performed with an assessment of the thromboembolic risk according to the Khorana score. In this analysis, approximately 12% of patients were defined as high-risk (score ≥ 3). In this subgroup, the incidence of VTE was 11.1% in the placebo arm and 4.5% in the nadroparin arm; thus, the NNT to prevent an event was 15, a significant reduction compared with the total study population where the NNT was 53. The benefit of prophylaxis was minimal in low-risk patients (NNT 77) [22]. Similarly, in a subgroup analysis of SAVE-ONCO the risk reduction was greater in high-risk patients (score ≥ 3) (5.4% for placebo vs. 1.4% for semuloparin) towards low-risk patients (score 0) (1.3% vs. 1%, respectively). No statistically significant difference was found in the incidence of major bleeding [23]. 

The question is still open for antithrombotic prophylaxis in cancer patients. On February 2019, two studies were published on antithrombotic prophylaxis with apixaban and rivaroxaban in cancer patients who were at high thromboembolic risk according to the Khorana score (≥2) and who are undergoing chemotherapy (AVERT and CASSINI) [24,25]. When considered together, the two trials showed a significant benefit of direct oral anticoagulants (DOACs) for VTE prophylaxis, with a low incidence of major bleeding [26]. A meta-analysis of randomized controlled trials on the prevention of VTE in ambulatory cancer patients treated with chemotherapy included also the Avert and Cassini studies and a phase II study with apixaban. Anticoagulant prophylaxis reduced the incidence of VTE by 49% (95% CI 0.43–0.61). The reduction in VTE was confirmed by limiting the analysis to the three studies with DOACs (OR 0.49; 95% CI 0.33–0.74). A significant increase in major bleeding was not observed in the analysis including all studies (OR 1.30, 95% CI 0.98–1.73). The reduction in VTE was confirmed in patients with lung cancer (OR 0.42, 95% CI 0.26–0.67), pancreatic cancer (OR 0.26; 95% CI 0.14–0.48) and in those estimated to be at high risk [16].

Several guidelines recommend or suggest primary prophylaxis with DOACs (rivaroxaban or apixaban) or LMWHs in outpatients with cancer at intermediate-to-high risk of VTE who are receiving systemic anticancer therapy and who are not actively bleeding or not at a high risk of bleeding [5,15,20,27]. Classification of patients into different risk classes should be based on a validated risk-assessment tool along with clinical judgment and experience [20]. According to some guidelines, this validated risk-assessment tool could be the Khorana score, and a threshold ≥2 can be considered for patients who can be prescribed antithrombotic prophylaxis [5,15]. Instead, the use of anticoagulation for routine prophylaxis of catheter-related thrombosis is not recommended [15,20], although peripherally inserted central catheters (PICCs) are associated with a higher risk of deep vein thrombosis (DVT) than centrally inserted central venous catheters, especially in cancer patients [28,29]. 

#### 2.1.2. What Can We Do?

It is important to identify cancer patients at a high risk of VTE who may benefit from drug prophylaxis to prevent VTE and to reduce the morbidity and mortality associated with VTE. There is a need to develop practical, realistic and useful risk assessment tools, which should be able to stratify cancer patients into high-, intermediate- and low-risk categories so that antithrombotic prophylaxis can be instituted in selected patients. DOACs are promising, but more studies are needed to consolidate future recommendations on the use of DOACs in this population of patients.

Outpatient cancer patients are the population of patients on which the application of antithrombotic prophylaxis is more difficult for various reasons:

1. They are a very heterogeneous population and the tools for stratifying thromboembolic risk are still imperfect;
2. DOACs are still not being used for prevention of VTE, despite the publication of important studies such as AVERT and CASSINI;
3. Oncologists still lack some confidence in the use of anticoagulants and there is still little collaboration with thrombosis experts.

Holmes and colleagues developed a model of care in their outpatient oncology clinic (Venous Thromboembolism Prevention in the Ambulatory Cancer Clinic Program), with the goal of increasing adherence to guidelines. The actions carried out were patient education about VTE risk, standardized VTE risk assessment and selection of patients at high risk for VTE for appropriate anticoagulation prophylaxis (drug, dose and duration). This model included a definite role for oncologists, nurses, oncology advanced practice providers, hematologists or thrombosis physician specialists, pharmacists and an electronic health record. High-risk patients discussed VTE prophylaxis options with the specialists. The risk of VTE at 6 months decreased by 12.8% in the implementation phase to 8.2% after implementation. This multidisciplinary approach was proven as effective in the assessment of VTE risk and in the selection of patient candidates for anticoagulation prophylaxis, with an effective reduction of VTE events [30].

Another effective strategy is to incorporate risk stratification tools into electronic medical records, with the aim of increasing awareness of CAT and alerting healthcare providers to consider VTE prophylaxis or objective investigations for patients at a high risk of VTE [31].

### 2.2. Treatment of CAT

#### 2.2.1. What Is the Evidence?

For many years, LMWHs have been considered the standard of care for VTE in cancer patients [32,33,34]. In recent years, DOACs have proven to be just as effective and safe as LMWHs [35,36,37,38,39,40]. LMWHs are very manageable for the ease of dose adjustment based on patient weight or particular clinical situations (e.g., thrombocytopenia) and the absence of drug–drug interactions. However, prolonged drug administration through subcutaneous injection is uncomfortable. Five trials, the Hokusai VTE Cancer trial, SELECT-D, the CASTA DIVA trial, the Caravaggio study and the ADAM VTE trial, directly compared DOACs, such as edoxaban, rivaroxaban and apixaban, respectively, to dalteparin for the treatment of CAT [41,42,43,44,45]. In all trials, DOACs were shown to be as effective and safe as dalteparin. A meta-analysis of aggregated efficacy and safety outcomes of these DOACs vs dalteparin showed a less frequent recurrent VTE with DOACs, but an increased risk of bleeding, especially in patients with gastrointestinal cancer [46]. A recent review confirmed DOACs as a standard of care for the treatment of CAT and showed that apixaban is particularly safe in patients with a high risk of bleeding [47].

Based on the results of all studies, international guidelines suggest DOACs (apixaban, edoxaban or rivaroxaban) over LMWHs or over VKAs for the treatment of VTE in patients with active cancer, and apixaban or LMWHs in patients with gastrointestinal cancer [20,48].

The role of endovascular therapy in cancer patients is controversial. Unfortunately, there are no prospective trials in cancer patients. In 2020, however, a retrospective study was published on several patients with cancer and lower-extremity proximal DVT undergoing catheter-directed thrombolysis compared with other patients treated with anticoagulation alone. No significant difference in mortality was found between the two groups (2.6% vs. 1.9%, *p* = 0.23), but =0·23), but the risk of intracranial bleeding was increased with thrombolytic therapy (1.3% vs. 0.4%, *p* = 0.017) [49].

NCCN guidelines suggest to consider catheter-directed therapy (pharmacomechanical thrombolysis or mechanical thrombectomy) in “patients at risk of limb loss (e.g., phlegmasia cerulea dolens), patients who demonstrate central thrombus propagation in spite of anticoagulation, and those with moderate to severely symptomatic proximal DVT” and particularly percutaneous mechanical thrombectomy in “patients with high bleeding risk or contraindication to thrombolytic therapy” [50]. These procedures should be decided on a case-by-case basis, paying attention to contraindications (bleeding risk), and should be performed in highly experienced centers.

One of the unmet clinical needs is the optimal duration of anticoagulant treatment in cancer patients with VTE. There is sufficient evidence to recommend anticoagulant treatment for at least 3–6 months [5,15,20,48], while there is still little evidence on the extension of anticoagulation beyond the first 3–6 months [51,52]. Risks and benefits of anticoagulant therapy beyond 6 months in patients with cancer are not yet well established. It appears that patients with active cancer, particularly if receiving anticancer treatment, can benefit from an extension of anticoagulant therapy, as long as they are at a low bleeding risk [5,15]. It is important to consider the risk of bleeding, life expectancy, cost of therapy and patient preference. The single-arm DALTECAN trial considered extended treatment with dalteparin on 334 cancer patients with VTE; among them 109 completed 12 months of dalteparin. The risk of major bleeding and of recurrent VTE was the greatest during the first month of treatment [51]. LMWH beyond 6 months was also evaluated in the single-arm TiCAT trial for the evaluation of tinzaparin on 247 patients enrolled; among them 136 completed 12 months of therapy, with a rate of clinically relevant bleeding 0.9% per patient–month during months 1 through 6 and 0.6% during months 7 through 12 [52]. The oral administration is often preferred in extended anticoagulant therapy. The choice between DOAC and LMWH should consider bleeding risk factors, such as unresected mucosal tumors, active mucosal lesions, use of antiplatelet agents, renal or hepatic impairment, thrombocytopenia, history of GI bleeding and drug–drug interaction. A study with two apixaban doses (5 mg twice daily or 2.5 mg twice daily) in patients with active cancer and VTE after 6 months of anticoagulant therapy with LMWH is ongoing (APICAT, NCT03692065) [53].

In cancer patients with recurrent VTE despite appropriate anticoagulant therapy, the following treatment options are suggested by the experts:

1. Increasing the LMWH dose by 20–25% or switching to DOACs;
2. Switching to LMWH for DOACs;
3. Switching to LMWH or DOACs for vitamin K antagonists;
4. Inserting an inferior vena cava (IVC) filter in combination with anticoagulation therapy in the case of pulmonary embolism (PE) [15].

The insertion of an IVC filter should also be considered in patients with an absolute contraindication to anticoagulant therapy. VTE treatment with once-daily LMWH is recommended for the initial treatment of VTE. Fondaparinux is a valid alternative to LMWH. Unfractionated heparin is an option for patients with severe renal insufficiency [15].

Thrombocytopenia is common in patients with cancer, especially in those with hematologic malignancies. It is related to the underlying disease or to antineoplastic therapy [54] and is associated with VTE recurrence in 27% of patients and with bleeding in 15% [55]. Randomized trials on anticoagulants in VTE excluded patients with marked thrombocytopenia (platelet count < 75 × 10^9^/L or 50 × 10^9^/L). Due to the lack of solid evidence from the literature, the appropriate management of acute VTE and thrombocytopenia is currently uncertain. In the real world, there is a tendency to use a reduced dose of LMWH, in some cases associated to platelet transfusions. In a recent prospective multicenter cohort study on 121 patients with CAT and thrombocytopenia, treated or with full-dose anticoagulation or with modified-dose anticoagulation, a lower rate of major hemorrhage and no recurrent VTE were observed with a modified-dose anticoagulation [56]. Currently, we can use a full therapeutic dose of anticoagulant drug (LMWH or DOAC) in cancer patients with acute VTE and a platelet count above 50 × 10^9^/L. If the platelet count is below 50 × 10^9^/L, we should take into account the risk of bleeding and VTE recurrence and evaluate case-by-case. We can use a reduced dose of LMWH if the platelet count is between 25 × 10^9^/L and 50 × 10^9^/L. For the use of DOACs in this scenery, we have data neither from studies nor from the real world. Instead, we can use neither LMWH nor DOACs in those with a platelet count of below 25 × 10^9^/L [15,57,58].

Patients with primary or metastatic brain cancer represent an issue for clinicians, who fear the onset of intracranial hemorrhages with anticoagulation. In a meta-analysis of nine retrospective studies, the risk of intracranial hemorrhage was two times higher in anticoagulated patients than in those without anticoagulation, and more than three times higher in patients with glioma, while for patients with brain metastases anticoagulation was not associated with an increased risk of intracranial hemorrhage [59]. A more recent meta-analysis confirmed an increased risk of intracranial hemorrhage (OR 3.66, 95% CI 1.84–7.29) in patients with glioma taking anticoagulants for VTE [60].

Limited data are available on the safety of DOACs in patients with brain cancer. Unfortunately, in the Hokusai-VTE Cancer study and in the SELECT-D study, few patients with brain cancer were included [41,42], while in the Caravaggio study those patients were not included [44]. In a retrospective cohort study, the cumulative incidence of intracranial hemorrhage was evaluated in 125 patients with primary and metastatic brain tumors on anticoagulation. The rate of major bleeding was 26% in the LMWH group versus 9.6% in the DOAC group (*p* = 0.03). The rate of intracranial hemorrhage was 15% in the LMWH group versus 5.8% in the DOAC group (*p* = 0.09). [61]. These results and findings from other retrospective cohort studies show that DOAC use in patients with brain tumors is as safe and effective as LMWHs.

Nowadays several central venous access devices are available, including implantable ports and PICCs. The risk of venous thrombosis is seven times higher in cancer patients than in patients without cancer [62]. The risk increases if a foreign intravascular body is present. Therefore, a venous thrombosis can commonly arise in patients with venous catheters, with an incidence of 14–18% of symptomatic forms and 5% of symptomatic forms [63].

The routine administration of pharmacologic prophylaxis to prevent central venous catheters thrombosis (CVCT) is not routinely recommended due to the lack of evidence of a benefit [64]. Optimal long-term anticoagulant treatment has not been established. In prospective studies, oral anticoagulation is typically administered for 3–6 months and as long as the catheter remains in place with favorable outcomes [64].

The removal is only necessary in cases of an incorrectly placed catheter, sepsis or a failed line [64]. The long-term administration of LMWH versus warfarin in cancer patients is recommended by current guidelines [64].

There is increasing evidence for DOACs in CAT, but there is still little data for the use of these agents in CVCT. A retrospective analysis of 83 patients with CVCT treated with rivaroxaban showed the efficacy and safety of the DOAC, with a few catheter removals (3.6%) and a few major bleedings (2.4%) [65]. A prospective study on 70 cancer patients with CVCT treated with rivaroxaban showed that the line function at 3 months was preserved in all patients; however, one patient died of PE and nine patients had bleeding. Therefore, rivaroxaban looks promising to treat CVCT in cancer patients given the preserved line function but requires further study before being recommended given the fatal PE episode and the excess bleeding [66]. A comparison study between rivaroxaban (20 mg/day) and LMWH/VKAs in the treatment of PICC-associated thrombosis showed a better efficacy profile with rivaroxaban compared with LMWH/VKAs, due to the faster resolution of thrombosis, and the same excellent safety profile [67].

For cancer patients with CVCT and a high risk of bleeding, catheter removal alone without anticoagulation is a viable option, as shown in a recent retrospective study comparing PICC removal alone versus PICC removal plus anticoagulation. A lower risk of major bleeding was found with catheter removal alone but at the price of a few more thromboembolic events [68].

Thrombosis of the portal, mesenteric or splenic veins, defined as splanchnic vein thrombosis (SVT), may be symptomatic or asymptomatic. In the latter case it is detected incidentally. Liver, pancreatic cancer and myeloproliferative diseases are at a higher risk of SVT. The presence of SVT in patients with liver and pancreatic cancer is associated with reduced survival [69]. Data on DOACs are derived from a registry of VTE patients by the Mayo Clinic [70] and from the randomized ADAM VTE trial [45]. The first study enrolled 48 patients with SVT patients, 26 of whom were treated with DOACs; 54% had an underlying malignancy. Recurrence rates and bleeding rates were similar between these patients and other patients who received enoxaparin (n = 22). All recurrence occurred in patients with underlying cancers. Similar results were found in the ADAM VTE trial, which enrolled 39 patients with SVT (8.2% of apixaban and 18.2% of dalteparin) [45]. ASH guidelines suggest treating patients with cancer-associated acute SVT with short-term anticoagulation or observing [20].

Approximately 50% of cancer-related PE events are incidentally diagnosed on routine computed tomography scans [71,72]. A meta-analysis of three recent RCTs comparing DOAC with LMWH (SELECT-D, Hokusai VTE Cancer and Caravaggio) and twenty observational studies, showed a significantly lower rate of recurrent VTE at 6 months in patients with incidental VTE compared with those with symptomatic VTE (relative risk (RR), 0.62; 95% CI, 0.44–0.87), but a non-significantly higher 6-month risk of major bleedings (RR, 1.47; 95% CI, 0.99–2.20) and the same overall mortality [72]. Patients with incidental PE may be asymptomatic or symptomatic. In a prospective cohort study of 695 patients with cancer-associated incidental PE signs, symptoms of PE had been present within 14 days before incidental PE diagnosis in 44% of the cases [73]. About 774 patients with incidental VTE were enrolled in RCTs on DOACs (SELECT-D, Hokusai VTE Cancer and Caravaggio). The risk of recurrent VTE was non-significantly lower in the DOAC group than in the LMWH group (RR 0.54; 95% CI 0.26–1.11), while the risk of major bleeding was non-significantly higher in patients with incidental VTE receiving DOACs than in those given LMWH (RR 1.29%; 95% CI 0.74–2.28) [74]. Current guidelines suggest therapeutic anticoagulation for cancer patients with incidental PE, while there is still uncertainty whether to treat patients with isolated subsegmental PE [5,20,48].

Antineoplastic drugs, in addition to being an independent risk factor for VTE in cancer patients, can interact with DOACs for inhibition or induction of P-glycoprotein and inhibition or induction of cytochrome P3A4 [75]. There is currently a lack of evidence regarding such interactions that often derive from studies in animals or in healthy volunteers. Thus, we still do not know the clinical impact of such interactions, until pharmacokinetic, pharmacodynamic and clinical outcome studies are available. Therefore, while waiting for information on the impact of cancer drugs on the efficacy and safety of DOACs, the collaboration between oncologists and vascular physicians/angiologist is important. 

**Figure 1:**
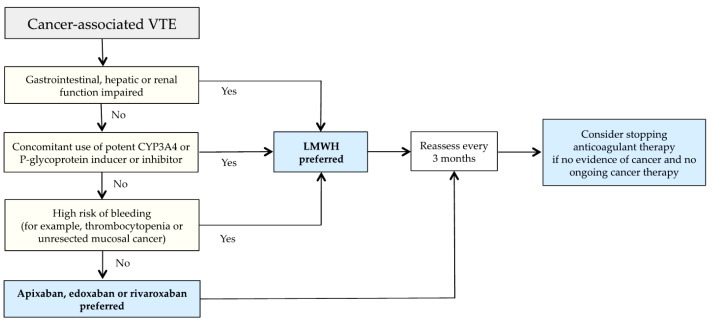
Clinical algorithm for management of cancer-associated VTE (modified by ref. [76]). VTE—venous thromboembolism; LMWH—low-molecular-weight heparin.

Clinical algorithms, which simplify the approach to the patient with CAT, are reported in the literature and should always take into account patient preferences. One of these clinical algorithms to individualize treatment of CAT, incorporating the risk of bleeding and drug–drug interactions, was recently proposed by Khorana and colleagues (Figure 1) [76].

**Figure 2:**
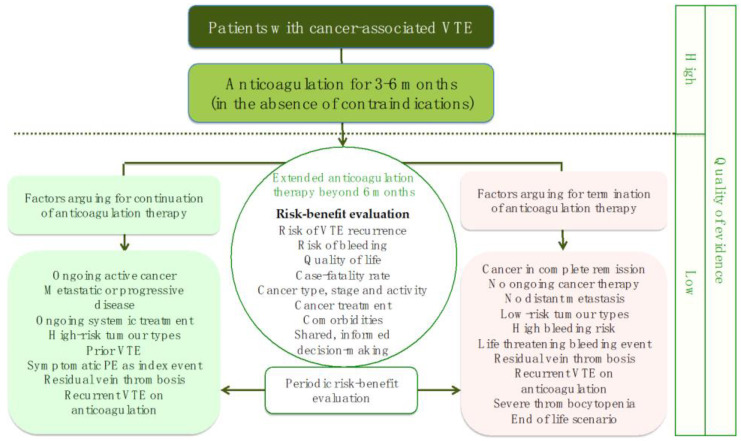
Extended anticoagulation therapy beyond six months (modified by ref. [77]). VTE—venous thromboembolism.

Another interesting algorithm recently proposed by Sanfilippo and colleagues regards extended anticoagulation therapy beyond six months (Figure 2) [77].

#### 2.2.2. What Can We Do?

One of the challenges of the treatment of VTE in cancer patients is the higher risk of VTE recurrence or bleeding under anticoagulants. Guidelines help physicians to harmonize practices, facilitating decision making in many settings. Unfortunately, cancer patients often have comorbidities and undergo complex treatment protocols, which may affect the efficacy and safety of anticoagulant treatments.

In such situations, multidisciplinary team meetings, involving specialists from different backgrounds (the vascular physician/angiologist in particular) could result in consensus-based decisions that improve outcomes. A French study, evaluating the impact of multidisciplinary team meetings on the management of VTE, has shown that such meetings are able to apply good clinical practice according to the guidelines on the standards of care [78]. A similar approach was confirmed to be associated with improved adherence to guidelines [79]. Integrated care pathways (ICPs) are defined as a methodology for the mutual decision making and organization of care for a well-defined group of patients in a well-defined period. Given its complexity, CAT often requires a multidisciplinary approach, very relevant for improving the quality of care. We should be encouraged to develop and implement ICPs on CAT. 

Other important aspects are the awareness of VTE in cancer patients, their adherence to anticoagulant treatment and quality of life.

Awareness of signs and symptoms of VTE is particularly low and treating physicians are often not effective in educating their patients about CAT [80]. Cancer patients have usually a low awareness of their risk of thrombosis. A survey on patient awareness and knowledge about CAT was carried out in Europe (Spain, UK, Germany, Greece, Italy, France) by the European Cancer Patient Coalition. A total of 1,365 participants (cancer patients or caregivers) were asked about risk factors, signs and symptoms and interventions. This survey found that only 28% of respondents were aware of their higher risk to develop VTE [81]. This inevitably leads to a delay in diagnosis and treatment. Patients should be more aware of CAT and seek treatment quickly, but health professionals should also be more aware of the problem. In fact, there are even knowledge deficits regarding CAT among healthcare providers (physicians, oncology nurses, oncologists) [31]. Educational strategies directed at nurses, hematologists and oncologists have shown an improvement in the nurse practice and in the ability to identify appropriate prophylaxis and treatment of CAT [31].

Nurses, who are in closer and longer contact with patients, can play an important role in patient education on the signs and symptoms of VTE. They could provide educational material and accept feedback from them. Fortunately, there has been an increase in the awareness among oncologists in Europe during the last years, but there is still a lot to do. There should be clinicians who are experienced in thrombosis in oncology units. 

Awareness improves adherence to the proposed treatments. Therefore, a patient education program dedicated to CAT is feasible and desirable to improve cancer patient empowerment, compliance with CAT treatment and quality of life [82]. Adherence to treatment and the quality of life of patients with cancer and VTE are impaired, especially if patients are treated with LMWHs. VTE has a negative impact on cancer patients’ quality of life, as demonstrated by the use of questionnaires on the quality of life [83]. Experiencing VTE can cause long-term psychological distress, also termed “post thrombotic panic syndrome” [84]. A recent study carried out in Spain has confirmed that patients with VTE had an impaired quality of life, but also showed that their increased distress correlated with better adherence to therapy [85]. Treating physicians would also take these aspects into account and would develop strategies to improve adherence to anticoagulant therapy for cancer patients with VTE.

Regarding the follow-up strategies and the duration of anticoagulation therapy, good clinical practice foresees a first control at 3 months or earlier if necessary (e.g., at 1 month). Subsequent angiological checks should be at 6 months and 12 months. Two important aspects for the angiologist/vascular physician are: to evaluate the possible recanalization of the veins by echo color Doppler (in the case of venous thrombosis) and to check for possible resolution of pulmonary hypertension by echocardiogram, if present at the index event (in the case of PE). In the latter case, it may be sufficient to ask whether there is dyspnea on exertion. The patient often undergoes control staging CT for cancer. Although this is not aimed at this purpose, it allows to ascertain the possible recanalization of the pulmonary arteries. The other important aspects to check are adherence to therapy, the search for new thromboembolic events and bleedings (even minor), the presence of other side effects and the control of all the drugs taken [75]. We must try to assess and minimize modifiable risk factors for bleeding (e.g., uncontrolled hypertension or other medication predisposing for bleeding such as NSAIDs) [75]. Finally, a sample of blood sampling (including hemoglobin, renal and liver function) should be collected [75]. The two specialists, such as the oncologist and the angiologist/vascular physician, should discuss the patients’ problems arising during the follow-up. Discontinuation of therapy should be done if the neoplasm is no longer in the active phase and/or anticancer therapy is not in progress. This is information that the oncologist knows well and that should be clear in case of evaluation of the patient by the angiologist/vascular physician. Possible dose reductions of anticoagulant therapy should always be discussed between the two specialists.

A multidisciplinary team with various specialists, in particular the oncologist and the angiologist/vascular physician, should jointly lead the management of the CAT patient and jointly manage complications, complexities and unmet needs. In fact, the follow-up following the initial decision-making moment is of paramount importance. One of the crucial points to be managed together by a multidisciplinary team is the duration of therapy, when to discontinue treatment in relation to the status of the neoplastic disease and the interference of drugs that may change during follow-up. A multidisciplinary discussion, and involvement of the patient to consider his/her needs, is crucial for the efficacy and safety of treatment.

## 3. Conclusions and Future Directions

Cancer is not always a one-time event. Cancer can become a chronic illness that may be controlled with treatments. VTE represents a complication for cancer patients that could itself begin a chronic health problem to manage. Firstly, VTE in cancer patients represents a therapeutic target, but after the first 3–6 months after the acute event (DVT and/or PE), the risk of VTE recurrence should be considered as a type of chronic disease to prevent as much as cancer is ongoing. Physicians, nurses, patients and caregivers should have this awareness.

The establishment of a multidisciplinary team, including an expert of vascular medicine, is mandatory for the better management of venous thromboembolic disease through periodic meetings.

A model of care in the outpatient oncology clinic should be instituted, including oncologists, primary oncology nurses, oncology advanced practice providers, hematologists or thrombosis physician specialists (vascular physicians/angiologists) and pharmacists. The objectives are education of cancer patients about VTE risk, periodic standardized VTE risk assessment, identification of patients at high risk for VTE, early diagnosis of VTE, prompt and adequate anticoagulant therapy with a favorable risk-benefit ratio, patient compliance and awareness, adherence to updated guidelines and development and implementation of integrated care pathways.

## Figures and Tables

**Table 1:** Khorana risk score.

Patient Characteristics	Score	OR (95% CI)
Site of cancer Very high risk (stomach, pancreas) High risk (lung, lymphoma, gynecologic, bladder, testicular) Other	2 1 0	4.3 (1.2–15.6) 1.5 (0.9–2.7)
Pre-chemotherapy platelet count ≥ 350 × 10^9^/L	1	1.8 (1.1–3.2)
Hemoglobin level < 10.0 g/dl or using red blood cell growth factors	1	2.4 (1.4–4.2)
Pre-chemotherapy leukocyte count > 11 × 10^9^/l	1	2.2 (1.2–4.0)
BMI ≥ 35 Kg/m^2^	1	2.5 (1.3–4.7)

BMI—body mass index. Cut-off scores: high risk, ≥3; intermediate risk, 1–2; low risk, 0.

## Data Availability

Data sharing not applicable.
